# Small-Molecule Oral Versus Injectable Glucagon-Like Peptide-1 (GLP-1) Receptor Agonists: Comparative Efficacy, Safety, and Future Clinical Perspectives

**DOI:** 10.7759/cureus.107202

**Published:** 2026-04-16

**Authors:** Digantkumar Patel

**Affiliations:** 1 Department of Hospital Medicine, Springfield Clinic, Springfield, USA

**Keywords:** cardiovascular outcomes, glp-1 receptor agonist, obesity, oral incretin, orforglipron, semaglutide, small molecule, tolerability, type 2 diabetes

## Abstract

Glucagon-like peptide-1 receptor agonists (GLP-1RAs) have transformed the management of type 2 diabetes (T2D), obesity, and cardiometabolic disease by improving glycemic control, promoting clinically meaningful weight loss, and reducing major adverse cardiovascular events (MACE) in selected populations. Historically, GLP-1RA therapy has relied on injectable peptide agonists (e.g., liraglutide, semaglutide, dulaglutide, and exenatide), which mimic native incretin biology but require parenteral administration and cold-chain logistics. In parallel, oral GLP-1RAs have emerged through two distinct strategies: (1) the oral delivery of peptide agonists using absorption enhancers (e.g., oral semaglutide) and (2) true small-molecule, non-peptide GLP-1 receptor agonists (e.g., orforglipron), designed to be orally bioavailable without peptide constraints. This narrative review compares small-molecule oral GLP-1RAs to injectable peptide agonists across efficacy (glycated hemoglobin {HbA1c} lowering, weight reduction, and cardiometabolic outcomes), safety and tolerability (gastrointestinal {GI} adverse events, gallbladder disease, pancreatitis signals, retinopathy considerations, and rare hepatic signals), real-world adherence, and future innovation. Recent phase 3 evidence suggests that oral small-molecule GLP-1RAs can deliver glycemic and weight benefits approaching injectable standards, while high-dose oral peptide formulations may broaden oral options for obesity management. Remaining challenges include long-term outcome data, the optimization of titration to improve tolerability, and equitable access amid rapid market expansion.

## Introduction and background

Glucagon-like peptide-1 (GLP-1) is a gut-derived incretin hormone secreted from intestinal L-cells in response to nutrient intake and plays a central role in glucose and energy homeostasis. Its physiological actions include the glucose-dependent stimulation of insulin secretion, suppression of inappropriate glucagon release, delay of gastric emptying, and promotion of central satiety signals. Through these complementary mechanisms, GLP-1 improves postprandial and fasting glycemic control while simultaneously reducing caloric intake and body weight. These pleiotropic metabolic effects have positioned GLP-1 receptor signaling as a cornerstone therapeutic target for type 2 diabetes (T2D) and obesity, two interrelated conditions that contribute substantially to global cardiometabolic morbidity and mortality.

Injectable peptide-based glucagon-like peptide-1 receptor agonists (GLP-1RAs) were the first clinically successful pharmacologic agents to exploit this pathway. Structural modifications of the native GLP-1 peptide have enabled resistance to enzymatic degradation and prolonged half-life, allowing once-daily or once-weekly subcutaneous administration. Over the past decade, multiple injectable GLP-1RAs have demonstrated robust reductions in glycated hemoglobin (HbA1c), clinically meaningful and sustained weight loss, and favorable effects on cardiometabolic risk factors. Importantly, large cardiovascular (CV) outcome trials have shown that several injectable GLP-1RAs reduce major adverse cardiovascular events (MACE) in patients with T2D and established or high cardiovascular risk, solidifying their role beyond glycemic control and into cardiovascular risk modification.

Despite their proven efficacy, the widespread uptake of injectable GLP-1RAs has been limited by practical and behavioral barriers, including needle aversion, injection training requirements, and concerns regarding long-term adherence. These challenges have driven sustained interest in developing orally administered GLP-1RA therapies, which offer the potential advantages of improved patient acceptance, simplified administration, and broader scalability in clinical practice. An effective oral GLP-1RA could significantly expand access to incretin-based therapy, particularly in earlier stages of disease and in populations reluctant to initiate injectable treatments.

However, the oral delivery of GLP-1 receptor agonists presents substantial pharmacologic challenges. Peptide-based agents are inherently susceptible to degradation by gastric and intestinal proteases and exhibit extremely low permeability across the gastrointestinal (GI) epithelium. These limitations initially rendered oral GLP-1 therapy impractical. Advances in drug-delivery science led to the development of peptide formulations co-administered with absorption enhancers, enabling localized gastric uptake and paving the way for the first oral peptide GLP-1RA. In parallel, medicinal chemistry efforts have focused on the development of orally bioavailable, non-peptide small-molecule GLP-1RAs that activate the receptor through alternative binding sites and signaling conformations.

The emergence of these distinct oral strategies, peptide-based formulations relying on absorption enhancers and true non-peptide small-molecule agonists, has introduced a new and rapidly evolving therapeutic landscape. While injectable peptide GLP-1RAs remain the most extensively validated agents with mature cardiovascular outcome data, oral GLP-1RA platforms raise important questions regarding comparative efficacy, safety, the durability of response, and long-term clinical impact. A comprehensive comparison of these therapeutic approaches is therefore essential to inform evidence-based treatment selection and to anticipate the future direction of incretin-based therapies.

This article was conducted as a narrative review aimed at synthesizing emerging evidence on oral small-molecule GLP-1 receptor agonists and comparing them to established injectable peptide agonists. A structured literature search was performed using PubMed/Medical Literature Analysis and Retrieval System Online (MEDLINE), Embase, and Google Scholar to identify relevant studies published between January 2015 and February 2026. Search terms included combinations of “GLP-1 receptor agonists,” “oral GLP-1,” “small-molecule GLP-1R agonist,” “orforglipron,” “injectable GLP-1 agonists,” “semaglutide,” “tirzepatide,” “liraglutide,” “weight loss,” and “type 2 diabetes.” Priority was given to randomized controlled trials, phase 2 and phase 3 clinical trials, meta-analyses, and major guideline statements. Additional relevant references were identified through the citation tracking of key articles. As a narrative review, formal systematic review methods or Preferred Reporting Items for Systematic Reviews and Meta-Analyses (PRISMA)-guided study selection were not applied; instead, studies were selected based on relevance to efficacy, safety, and emerging therapeutic developments in GLP-1 receptor agonist pharmacology.

## Review

GLP-1 biology and therapeutic rationale

Endogenous GLP-1 is secreted from intestinal L-cells in response to nutrient ingestion and enhances glucose-dependent insulin secretion [1]. GLP-1 suppresses glucagon secretion during hyperglycemia, helping reduce hepatic glucose output [1,2]. GLP-1 slows gastric emptying, contributing to postprandial glucose lowering and early satiety [2,3]. GLP-1 signals in hypothalamic and brainstem pathways to reduce appetite and energy intake [3,4]. Native GLP-1 is rapidly degraded by dipeptidyl peptidase-4 (DPP-4), resulting in a short half-life that limits direct therapeutic use [5]. Pharmacologic GLP-1R activation yields pleiotropic metabolic effects, including improvements in blood pressure and lipids in many populations [6]. The glucose-dependent mechanism contributes to a relatively low intrinsic hypoglycemia risk when used without insulin or sulfonylureas [7]. The class has expanded beyond glucose control to weight management and cardiovascular risk modification in selected populations [8].

Defining the comparison: “Small-molecule oral GLP-1RAs” versus “injectable peptide agonists”

Injectable GLP-1RAs are peptides engineered to resist DPP-4 degradation and prolong half-life via albumin binding, fatty acid acylation, or Fc-fusion strategies [9]. Injectable peptide agonists include daily (e.g., liraglutide and lixisenatide) and weekly (e.g., semaglutide, dulaglutide, and exenatide extended-release {ER}) preparations [10]. Oral peptide GLP-1RA delivery (e.g., oral semaglutide) is not a small molecule; it uses absorption-enhancing technology to deliver a peptide through the stomach [11]. True small-molecule oral GLP-1RAs are non-peptide ligands designed to activate the GLP-1 receptor (a class B G protein-coupled receptor {GPCR}) through alternative binding interactions [12]. Small-molecule GLP-1RAs aim to avoid peptide limitations (enzymatic degradation and poor membrane permeability) and enable conventional oral dosing [12,13]. Because binding modes differ, small molecules may produce distinct signaling bias, receptor kinetics, and tolerability profiles compared to peptides [14]. Clinically, the key comparative questions involve efficacy magnitude, durability, safety, convenience, adherence, and cost-effectiveness under real-world constraints [15].

To orient readers to the fundamental differences between true small-molecule oral GLP-1 receptor agonists and injectable peptide-based agents, Table 1 summarizes key distinctions across molecular design, receptor engagement, delivery, and practical implementation* *[9-16].

**Table 1:** Key distinctions: small-molecule oral GLP-1RAs versus injectable peptide agonists GLP-1RA, glucagon-like peptide-1 receptor agonist; ECD, extracellular domain; PK, pharmacokinetics; DDI, drug-drug interaction; GI, gastrointestinal; CVOT, cardiovascular outcome trial; RCT, randomized controlled trial

Domain	Small-molecule oral GLP-1RAs (true non-peptide)	Injectable peptide GLP-1RAs (and incretin injectables)
Molecular class	Non-peptide ligands	Peptide agonists engineered for stability and prolonged exposure
Receptor engagement	Often transmembrane-pocket-binding; may differ in signaling kinetics/bias	Typical ECD + transmembrane engagement; high-potency canonical activation
Delivery route	Oral (subject to food effects, first-pass metabolism, and DDI potential)	Subcutaneous injection (bypasses GI degradation; more predictable exposure)
Dosing cadence (typical)	Usually daily (depends on agent/formulation)	Daily or weekly (many long-acting weekly options)
Exposure variability	Potentially higher interpatient variability (absorption/metabolism dependent)	Generally lower variability compared to oral dosing
Practical advantages	No injections; no devices; potentially reduced cold-chain constraints	Less frequent dosing (weekly options); predictable PK; established outcome evidence
Practical limitations	Adherence depends on daily routine; DDI/metabolic considerations	Injection aversion; device handling; cold storage considerations for some products
Evidence maturity	Emerging; outcome evidence still developing	Extensive RCT and CVOT evidence base for multiple agents

Pharmacology and receptor pharmacodynamics

The GLP-1 receptor is a class B GPCR with a large extracellular domain (ECD) that is central to peptide binding and activation [16]. Peptide agonists typically engage both the ECD and transmembrane domain, stabilizing active receptor conformations with high potency [16,17]. Many therapeutic peptides are designed to slow clearance, enabling once-weekly dosing with sustained exposure and stable receptor engagement [9,10]. Small molecules are generally designed to bind within the transmembrane region and may activate the receptor with different conformational fingerprints [12,14]. Differences in residence time at the receptor may affect efficacy and adverse events, especially nausea driven by central and peripheral pathways [18]. Gastric-emptying effects show tachyphylaxis over time for some GLP-1RAs, while central appetite effects may remain more durable [19]. Dose titration is used clinically to reduce nausea/vomiting and enhance persistence for both oral and injectable GLP-1RA approaches [20]. For oral therapies, absorption variability can influence exposure and clinical response more than with injectables, affecting dose-response interpretation [11,21].

Pharmacokinetics (PK) and delivery constraints

Injectable peptide GLP-1RAs bypass gastrointestinal degradation and provide predictable systemic exposure with relatively low interpatient variability [10]. Weekly injectables achieve long half-lives through molecular modifications (e.g., acylation and fusion proteins), promoting stable trough concentrations [9,10]. Oral peptide semaglutide relies on an absorption enhancer, sodium N-(8-[2-hydroxybenzoyl] amino) caprylate (SNAC), to facilitate transcellular uptake in the stomach and must be taken under strict fasting conditions [11]. Oral peptide absorption is low and variable, requiring higher oral doses than injectable equivalents to achieve therapeutic exposure [11,21]. Small-molecule oral GLP-1RAs are designed for improved permeability and stability, potentially reducing stringent administration requirements [12,13]. However, oral small molecules may still face food effects, first-pass metabolism, and drug-drug interactions via hepatic enzymes or transporters [22]. Peak-trough dynamics may be more pronounced with daily oral agents, potentially influencing tolerability and symptom timing [23]. Renal or hepatic impairment can differentially affect clearance depending on the molecule and metabolic pathway, requiring agent-specific guidance [24].

Key pharmacokinetic and delivery-related differences between injectable peptide and small-molecule oral GLP-1RAs are summarized in Table 2 [9-13,21,22,24].

**Table 2:** Pharmacokinetic and delivery differences between injectable peptide and small-molecule oral GLP-1 receptor agonists GLP-1RA, glucagon-like peptide-1 receptor agonist; GI, gastrointestinal

Feature	Injectable peptide GLP-1RAs	Small-molecule oral GLP-1RAs
Route of administration	Subcutaneous injection	Oral
Gastrointestinal degradation	Avoided due to parenteral delivery	Subject to gastrointestinal enzymatic degradation
First-pass hepatic metabolism	Absent	Present
Absorption predictability	High and consistent	Variable, influenced by food and GI physiology
Pharmacokinetic variability	Low interpatient variability	Higher interpatient variability
Half-life	Prolonged (hours to weeks and formulation-dependent)	Generally shorter (hours to approximately one day)
Dosing frequency	Daily or weekly (long-acting formulations available)	Typically daily
Peak-trough fluctuations	Relatively stable with long-acting agents	More pronounced due to oral dosing
Drug-drug interaction potential	Minimal	Greater potential via hepatic metabolism
Storage and handling	Injection devices; some formulations require cold storage	No injection devices; simplified storage and transport
Delivery scalability	Limited by injection logistics	Potentially broader due to oral administration

Evidence base for injectable peptide GLP-1RAs: Glycemic efficacy

In type 2 diabetes, injectable GLP-1RAs reduce HbA1c meaningfully, often in the range of ~0.8%-1.8% depending on baseline HbA1c, comparator, and dose [10,25]. Semaglutide demonstrated strong HbA1c reduction in the SUSTAIN program, with consistent superiority versus several active comparators [26]. Dulaglutide showed durable glycemic control across AWARD trials and broad real-world uptake due to weekly dosing and easy devices [27]. Liraglutide provided robust glycemic lowering with daily dosing and extensive experience across diverse patient phenotypes [28]. Exenatide ER and lixisenatide improved glycemia, with more pronounced postprandial effects often linked to gastric emptying [29]. Injectable GLP-1RAs are frequently positioned before insulin intensification in guidelines because of weight-loss advantages and lower hypoglycemia risk [30]. When combined with basal insulin, GLP-1RAs can reduce insulin needs and improve glycemia while mitigating weight gain [31]. Head-to-head trials generally show greater HbA1c and weight reductions with semaglutide compared with several other GLP-1RAs at labeled doses [25,26].

Evidence base for injectable peptide GLP-1RAs: Weight-loss efficacy

Weight loss occurs via reduced appetite, lower energy intake, and, particularly early in therapy, slower gastric emptying [3,19]. Liraglutide 3.0 mg demonstrated clinically meaningful weight loss in obesity trials and became an early GLP-1RA option for chronic weight management [32]. Semaglutide 2.4 mg produced substantial mean weight reductions in STEP trials, elevating GLP-1RA therapy to a new standard for obesity pharmacotherapy [33]. Tirzepatide, a dual gastric inhibitory polypeptide (GIP)/GLP-1 agonist administered by injection, achieved very large weight reductions in the SURMOUNT phase 3 obesity trial program [34] and strong glycemic control in SURPASS trials [35], often exceeding single-agonist GLP-1RAs. The weight-loss magnitude generally correlates with dose/exposure and may be limited by tolerability in some patients [20,33]. Weight regain after discontinuation is common, supporting long-term therapy models akin to hypertension or dyslipidemia management [36]. Real-world persistence strongly influences weight outcomes, making the route of administration and tolerability central to comparative effectiveness [37]. Combination with lifestyle interventions improves outcomes, but medication effect sizes remain clinically significant even with variable behavioral adherence [33,38].

Cardiovascular outcome evidence: Injectable GLP-1RAs

Several injectable GLP-1RAs have demonstrated cardiovascular benefit in large outcome trials in type 2 diabetes with high cardiovascular risk [39]. Liraglutide reduced major adverse cardiovascular events (MACE) in LEADER, supporting cardioprotective class positioning [40]. Semaglutide reduced MACE in SUSTAIN-6, with particular interest in stroke outcomes and retinopathy signals in rapid glycemic improvement contexts [41]. Dulaglutide reduced MACE in REWIND, notably including a large proportion of participants without established cardiovascular disease [42]. Albiglutide reduced MACE in HARMONY Outcomes, further supporting cardioprotection across multiple agents [43]. Lixisenatide demonstrated cardiovascular safety (non-inferiority) in ELIXA rather than superiority, highlighting heterogeneity in effect sizes across molecules and populations [44]. Mechanisms proposed include weight loss, blood pressure reduction, anti-inflammatory effects, improved endothelial function, and modest lipid improvements [45]. The presence of multiple positive trials has driven the guideline prioritization of GLP-1RAs for patients with diabetes and high cardiovascular risk [30,46].

Kidney outcomes and metabolic-renal protection

GLP-1RAs reduce albuminuria and may slow kidney function decline, though effect sizes and endpoints vary across trials [47]. In outcome trials, renal composite benefits have often been driven by reduced new macroalbuminuria [39,47]. Indirect benefits include improved blood pressure, weight, and glycemic control, all of which support kidney protection [45,47]. Comparatively, sodium-glucose cotransporter-2 (SGLT2) inhibitors have more consistent hard kidney endpoint data, but GLP-1RAs complement them in metabolic-renal care [48]. Combination therapy (GLP-1RA + SGLT2 inhibitor) is increasingly used for additive metabolic and cardiorenal benefits [49]. The practical implication is that easier access and adherence to GLP-1RA therapy (potentially via oral small molecules) could translate into broader population renal benefit [15,37]. Ongoing and future trials are expected to better define kidney-specific outcomes for newer incretin agents [50]. Agent selection in chronic kidney disease (CKD) often depends on labeling, tolerability, and clinical priorities (weight, HbA1c, and CV risk) [30].

Safety profile of injectable peptide GLP-1RAs

Gastrointestinal (GI) adverse effects (nausea, vomiting, diarrhea, and constipation) are the most common class-related events and are dose-dependent [20,51]. Slow titration reduces GI symptoms and improves persistence, emphasizing patient education as a core implementation strategy [20]. Gallbladder-related events (e.g., cholelithiasis and cholecystitis) occur more often than placebo in some trials, possibly related to rapid weight loss and biliary effects [52]. Pancreatitis risk has been debated; large trials and meta-analyses generally do not show a major increase, but caution is advised in patients with prior pancreatitis [53]. Rodent C-cell tumor findings led to contraindications in patients with personal/family history of medullary thyroid carcinoma or multiple endocrine neoplasia type 2 (MEN2) for most GLP-1RAs [54]. Hypoglycemia risk is low with monotherapy but increases when combined with insulin or sulfonylureas, requiring dose adjustments [7,31]. Diabetic retinopathy complications were observed in some semaglutide contexts, likely related to rapid HbA1c improvements in high-risk patients [41,55]. Rare events include injection site reactions and, for certain formulations, immunogenicity concerns that are generally clinically modest [10].

Practical limitations of injectable therapy influencing comparative value

Injection aversion, needle phobia, and stigma can reduce acceptance and persistence despite strong efficacy [56]. Cold-chain storage requirements and device handling may complicate the use for patients with unstable housing, travel needs, or limited health literacy [57]. Weekly dosing improves convenience but does not eliminate injection barriers, especially for patients with significant aversion [56]. Supply limitations and cost/coverage barriers can disrupt continuity, leading to discontinuation and weight regain [58]. Titration schedules require follow-up and coaching, creating operational burdens for clinics without dedicated medication management infrastructure [59]. Health systems increasingly use standardized workflows and multidisciplinary teams to improve tolerability management and adherence [59,60]. Shared decision-making is crucial to align expected benefits (weight, HbA1c, and CV risk) with tolerability and access realities [60]. These limitations provide the strongest rationale for oral alternatives that maintain efficacy while improving uptake [15,56].

True oral GLP-1 receptor agonism: From molecular design to clinical promise

Rationale for Small Molecules: Moving Beyond Peptide Delivery Challenges

Peptide drugs generally have poor oral bioavailability due to enzymatic degradation and low membrane permeability [61]. Oral peptide delivery can work but often requires absorption enhancers and strict administration rules that reduce real-world convenience [11,21]. Small molecules can be engineered for oral absorption, scalable manufacturing, and potentially lower distribution complexity [12,13]. Small molecules may allow broader global access by reducing cold-chain dependence and device costs [57,62]. Daily oral dosing may improve psychological acceptability for patients reluctant to inject for long term [56]. However, daily dosing can also worsen adherence compared with weekly injections for some patients, making net persistence uncertain [63]. A major goal is to achieve injectable-like efficacy with oral simplicity and manageable GI tolerability [15,64]. The success criteria also include cardiometabolic outcome evidence, not only short-term HbA1c and weight changes [39,65].

Mechanistic Distinctions: Binding, Signaling, and “Functional Selectivity”

The GLP-1 receptor accommodates peptide binding via the extracellular domain, but small molecules often target transmembrane pockets [16,12]. Small-molecule agonists may act as ago-allosteric ligands, stabilizing active conformations differently than peptides [14,66]. Signaling bias (favoring cyclic adenosine monophosphate {cAMP} versus β-arrestin pathways) could theoretically alter efficacy or adverse-event profiles [14,67]. Receptor internalization and desensitization kinetics differ by ligand and may influence chronic response [68]. Some ligands may produce potent glycemic effects with less gastric slowing, potentially improving tolerability [19,64]. Conversely, reduced gastric slowing might attenuate postprandial glucose benefits for certain patients [29,69]. Translating mechanistic distinctions into patient-level outcomes requires careful clinical trial design and head-to-head comparisons [25,70]. Mechanistic diversity also raises the possibility of combination or sequencing strategies within “GLP-1RA” therapy classes [71].

Clinical Development Landscape: Examples and Trial Design Considerations

Small-molecule oral GLP-1RAs have been evaluated in phase 1-3 programs with endpoints including HbA1c, weight, and tolerability [64,72]. Dose-finding is central because exposure-response curves differ from peptides and can be confounded by GI-driven discontinuation [20,73]. Trials often compare multiple dose arms against placebo and sometimes include active comparators (injectable GLP-1RA) to contextualize effect size [70,72]. GI adverse events remain a primary driver of dropout and dose limitations, similar to peptides but with possibly different temporal patterns [51,73]. Daily oral administration introduces adherence variability that can dilute observed efficacy in intention-to-treat analyses [63,74]. Food effects and administration constraints must be evaluated early, as they can determine feasibility for real-world scaling [22,75]. Because weight loss and glycemic outcomes can be large, background lifestyle interventions should be standardized to reduce noise [33,38]. Ultimately, cardiovascular outcome trials will be required to confirm whether small-molecule oral agents reproduce the cardioprotective effects seen with several injectable peptides [39,65].

Because currently available oral small-molecule GLP-1RA trials differ in population, treatment duration, dose-escalation strategy, and comparator design, cross-trial comparisons with injectable peptide GLP-1RAs should be interpreted cautiously; however, the emerging data suggest clinically meaningful glycemic and weight-loss efficacy, particularly for orforglipron, while long-term cardiovascular outcomes and post-marketing safety remain to be established (Table 3) [64-69].

**Table 3:** Summary of key clinical trial outcomes for emerging oral small-molecule GLP-1 receptor agonists Values represent placebo-adjusted changes unless otherwise specified. Cross-trial comparisons should be interpreted cautiously due to differences in study design, patient populations, and treatment duration *Oral semaglutide is an oral peptide GLP-1 receptor agonist and is included for contextual comparison; it is not a small-molecule GLP-1 receptor agonist GLP-1RA, glucagon-like peptide-1 receptor agonist; T2D, type 2 diabetes; HbA1c, glycated hemoglobin; GI, gastrointestinal; AEs, adverse events

Agent	Trial/phase	Population	Duration	Main efficacy	Safety/notes
Orforglipron	Phase 2	T2D	26 weeks	HbA1c up to -2.10%; weight up to -10.1 kg	Mostly GI AEs; efficacy exceeded placebo and was numerically greater than dulaglutide in this study
Orforglipron	ACHIEVE-1, phase 3	Early T2D	40 weeks	HbA1c: -1.24% to -1.48%; weight: -4.5% to -7.6%	GI AEs mostly during escalation; discontinuation: 4.4%-7.8%
Orforglipron	ATTAIN-1, phase 3	Obesity without diabetes	72 weeks	Weight: -7.5% to -11.2%	GI AEs mostly mild-moderate; discontinuation: 5.3%-10.3%
Danuglipron	Phase 2b	T2D	16 weeks	HbA1c placebo-adjusted up to -1.16%; weight placebo-adjusted up to -4.17 kg	GI AEs common; twice-daily dosing
Lotiglipron	Phase 2	T2D/obesity	16-20 weeks	HbA1c up to -1.44%; weight up to -7.47%	Transaminase elevations observed; development terminated
Oral semaglutide*	OASIS 1, phase 3	Obesity without diabetes	68 weeks	Weight -15.1% versus -2.4% placebo	Include only as an oral peptide comparator, not as a small molecule

Because emerging oral GLP-1RAs are often compared to established injectable agents across multiple clinical domains, Table 4 outlines a structured framework for interpreting efficacy, safety, and real-world effectiveness across therapeutic classes [20,30,33,36,37,39-42,47-49,58,74].

**Table 4:** Clinical outcome comparison framework (what “parity” versus “complement” looks like) HbA1c, glycated hemoglobin; MACE, major adverse cardiovascular events; eGFR, estimated glomerular filtration rate; CKD, chronic kidney disease; CV, cardiovascular; MI, myocardial infarction; HF, heart failure; SGLT2i, sodium-glucose cotransporter-2 inhibitor; GI, gastrointestinal; AEs, adverse events

Outcome domain	What to compare	Why it matters clinically	Key confounders in comparisons
HbA1c lowering	Mean change, responder rates (≥1% drop), and durability	Drives microvascular risk reduction and treatment escalation decisions	Baseline HbA1c, adherence, background therapy, and dose titration limits
Weight reduction	Percent weight loss (≥5%, ≥10%, and ≥15%) and durability	Central to cardiometabolic risk reduction and patient-driven goals	Lifestyle intensity, persistence, and discontinuation/weight regain
Cardiovascular benefit	MACE (CV death/MI/stroke) and HF events	Payer coverage and guideline prioritization depend on outcome evidence	Population risk level, trial duration, and endpoint definitions
Kidney benefit	Albuminuria, eGFR slope, and renal composites	Relevant for diabetes-CKD phenotype and long-term morbidity	Background SGLT2i use and baseline CKD stage
Safety/tolerability	GI AEs, gallbladder events, pancreatitis signals, and retinopathy concerns	Main driver of discontinuation and dose limitation	Titration speed, dose/exposure peaks, and comorbidity risk
Real-world persistence	Time on therapy and dose maintenance	Determines “effectiveness” versus “efficacy”	Access/cost, supply issues, and adverse-event management support

Glycemic Efficacy: What “Parity” Would Mean Clinically

Injectable GLP-1RAs set a high bar for HbA1c reduction, often exceeding many oral agents when adherence is maintained [10,25]. To be clinically interchangeable, a small-molecule oral GLP-1RA would ideally achieve comparable HbA1c lowering at tolerated doses across baseline HbA1c strata [15,64]. Demonstrating superiority versus standard oral therapies (metformin, DPP-4 inhibitors, and some insulin-sparing) is necessary but insufficient for parity claims versus injectables [30,76]. Many patients require combination therapy; thus, oral small-molecule GLP-1RAs must be evaluated in add-on settings, including with SGLT2 inhibitors and basal insulin [49,77]. A key advantage could be earlier initiation in patients unwilling to inject, improving lifetime glycemic exposure [56,78]. Earlier initiation may improve β-cell preservation indirectly by reducing glucotoxicity and weight-related insulin resistance [79]. In practice, moderate HbA1c efficacy with better uptake might outperform high-efficacy injectables that are never started or are quickly discontinued [37,56]. Therefore, comparative effectiveness must incorporate initiation rates, persistence, and dose maintenance, not only per-protocol efficacy [37,74].

Weight-Loss Efficacy: What to Compare Against

Injectable semaglutide 2.4 mg and tirzepatide have shifted expectations by producing large mean weight reductions in obesity trials [33,34]. A small-molecule oral GLP-1RA with modest weight loss may still be valuable if it enables broad access and long-term adherence [15,58]. Weight outcomes depend on dose escalation, tolerability, behavioral context, and treatment duration [20,33]. If daily oral small molecules yield smoother exposure or less severe peak-related nausea, they might support higher sustained dosing in some patients [23,64]. However, if daily dosing reduces adherence relative to weekly injections, realized weight loss may be smaller [63,74]. Weight loss is clinically meaningful even at 5%-10%, improving glycemia, blood pressure, fatty liver markers, and sleep apnea severity in many patients [80]. The “best” agent may differ by patient priority: maximal weight loss versus modest weight loss with easier delivery and coverage [60,81]. Future head-to-head studies between small-molecule oral GLP-1RAs and injectable peptides will be essential to define true comparative weight efficacy [70,82].

To synthesize the relative strength of evidence across major metabolic outcomes, Table 5 provides an evidence-weighted comparison of injectable peptide GLP-1RAs and emerging small-molecule oral agents [10,30,33-35,39-43,47-49,56-58,65].

**Table 5:** Expected benefits and relative strength of evidence by GLP-1RA therapeutic class GLP-1RA, glucagon-like peptide-1 receptor agonist; T2D, type 2 diabetes; CVOT, cardiovascular outcome trial; CV, cardiovascular; RCT, randomized controlled trial; MACE, major adverse cardiovascular event

Benefit	Injectable peptide GLP-1RAs	Small-molecule oral GLP-1RAs
HbA1c lowering (T2D)	Strong and consistent across multiple RCT programs	Emerging; magnitude and durability depend on dose/exposure and adherence
Weight loss (obesity/T2D)	Strong; high-efficacy benchmarks established (e.g., semaglutide 2.4 mg; incretin injectables)	Emerging; scalability could be high if tolerability/adherence is favorable
Cardiovascular outcomes	Multiple agents show MACE reduction (agent- and population-dependent)	Outcome trials needed to confirm CV benefit at scale
Kidney outcomes	Albuminuria reduction and favorable renal composites in CVOTs; often driven by macroalbuminuria endpoints	Unknown/early; likely needs outcome-level confirmation
Operational scalability	Limited by injections, devices, and some cold-chain constraints	Potentially high due to the oral route and simplified distribution
Overall evidence maturity	High (large CVOTs and long-term clinical experience)	Moderate to low currently (development stage and early trials dominate)

Safety and Tolerability: Similarities and Possible Differences

GI adverse events are likely to remain central with small-molecule GLP-1RAs because GLP-1 receptor activation drives nausea pathways [51,83]. The incidence and severity may differ based on receptor kinetics and signaling bias, but clinical confirmation is required [14,67]. Oral administration may shift symptom timing (e.g., nausea occurring after each dose) compared to weekly injections that have slower absorption profiles [23,84]. Hepatic metabolism introduces potential for drug-drug interactions that are generally less prominent with peptide injectables [22,85]. Because small molecules are structurally distinct, idiosyncratic toxicities (e.g., liver enzyme elevations) must be carefully monitored in development [86]. Class warnings related to thyroid C-cell tumors are based on GLP-1R biology and rodent findings, so labeling may still apply depending on regulatory decisions [54,87]. If small molecules produce less gastric slowing, they may reduce issues related to gastroparesis exacerbation but could also reduce postprandial glucose benefit [19,69]. As with all GLP-1R-based therapies, careful peri-procedural management may be needed due to effects on gastric emptying and aspiration risk considerations [88].

Although GLP-1 receptor activation confers class-wide adverse effects, formulation-specific considerations influence monitoring and risk mitigation. These similarities and differences are summarized in Table 6 [7,20,31,41,51-55,83-88].

**Table 6:** Safety and monitoring: shared class issues versus route-specific considerations GI, gastrointestinal; SU, sulfonylurea; GLP-1RAs, glucagon-like peptide-1 receptor agonists; HbA1c, glycated hemoglobin

Safety domain	Injectable peptide GLP-1RAs	Small-molecule oral GLP-1RAs
GI adverse effects (nausea/vomiting/diarrhea/constipation)	Common; dose-dependent; titration reduces dropout	Likely common; symptom timing may align with daily dosing peaks
Hypoglycemia (intrinsic)	Low unless combined with insulin/SU	Expected low unless combined with insulin/SU
Gallbladder disease	Increased risk observed in some contexts; weight-loss-related contribution	Theoretical risk likely similar if weight loss is significant
Pancreatitis	Overall risk not clearly increased in large analyses; caution in history	Similar caution expected; requires post-marketing confirmation
Thyroid C-cell tumor warning	Class labeling/contraindications apply for most GLP-1RAs	May apply depending on regulatory labeling and mechanism
Retinopathy considerations	Rapid HbA1c drops may worsen retinopathy in high-risk patients	Same concern if rapid glycemic improvement occurs
Drug-drug interactions	Minimal (peptides)	Higher potential via hepatic enzymes/transporters (agent-specific)
Peri-procedural gastric emptying	Aspiration risk discussions; evolving guidance	Similar concern if gastric emptying is affected; agent-specific

Adherence, Persistence, and Patient Experience: The “Real-World” Battleground

Medication persistence for chronic weight management and diabetes therapies is often low, driven by side effects, cost, and perceived benefit [37,58]. Oral administration may reduce injection-related barriers but does not eliminate GI intolerance or affordability constraints [56,58]. Daily dosing can be either helpful (habit formation) or harmful (missed doses), depending on patient routines and support systems [63,89]. Weekly injections reduce dosing frequency but can be disrupted by supply issues and may require device competence [57,58]. Patient education on titration, dietary strategies (smaller meals and lower fat), and symptom management are critical for both oral and injectable therapies [20,59]. Telehealth check-ins and pharmacist-led titration programs can improve persistence and reduce discontinuation [59,90]. Patient-reported outcomes, satiety, cravings, energy, and GI comfort, may differ by ligand and are increasingly important for comparative decision-making [81,91]. Therefore, the practical winner may be the therapy that maximizes long-term continuation at an effective dose within the patient’s access constraints [37,74].

Given that long-term benefit depends on persistence and patient acceptance, Table 7 outlines practical scenarios in which oral versus injectable GLP-1RA strategies may be preferentially selected [30,37,56-60,63,74,92].

**Table 7:** Practical implementation considerations when selecting oral versus injectable GLP-1RA therapy GLP-1RA, glucagon-like peptide-1 receptor agonist; CV, cardiovascular; MACE, major adverse cardiovascular events; CVOT, cardiovascular outcome trial; DDI, drug-drug interaction

Clinical scenario	Oral small-molecule GLP-1RA may be favored when…	Injectable peptide GLP-1RA may be favored when…
Injection reluctance/needle aversion	Patient strongly prefers oral therapy; injection barrier is the primary reason for non-initiation	Patient is willing to inject and prioritizes established outcome data
Need for maximal weight loss	Oral therapy improves the likelihood of starting and staying on therapy for long term	The highest efficacy is needed, and the patient can tolerate titration
Need for outcome-proven CV benefit	An oral agent has outcome evidence (future), or access is the only viable path	Established MACE reduction is desired with proven CVOT agents
Complex medication list	DDI risk is low/known and manageable	Avoids hepatic DDI concerns typical of small molecules
Variable routines/adherence issues	Patient can reliably take daily medications; reminders/support are available	Weekly dosing reduces pill burden and missed-dose risk
Limited storage/unstable environment	Oral route reduces device/cold-chain burden	Patient has reliable storage and can manage device use
Insurance/coverage constraints	Oral route is preferred, and formulary access is better	Injectable is covered, and supply is reliable

Comparative Implementation in Diabetes Care Pathways

Guidelines generally prioritize GLP-1RAs in patients with type 2 diabetes and established atherosclerotic cardiovascular disease (ASCVD) or high risk, independent of baseline HbA1c in some pathways [30,46]. Oral small-molecule GLP-1RAs could enable earlier adoption in primary care settings where injections are underused [78,92]. Earlier initiation may reduce therapeutic inertia, a major contributor to long-term complications in type 2 diabetes [93]. For patients needing large HbA1c reductions, injectable peptides (or dual agonists) may remain preferred until oral efficacy matches them [25,35]. For patients primarily seeking moderate HbA1c and weight benefit without injections, oral options may meaningfully expand treatment reach [15,56]. Combination with metformin and/or SGLT2 inhibitors is common, and future oral GLP-1RAs must be assessed in these multidrug contexts [49,77]. In insulin-treated patients, GLP-1RAs reduce insulin requirements and weight, but oral agents must prove comparable benefit and practicality [31,77]. Health system formularies and payer rules will strongly shape which pathway becomes dominant, regardless of pharmacology [58,94].

As therapeutic decision-making in type 2 diabetes increasingly prioritizes individualized care pathways, Table 8 summarizes key considerations influencing the selection of oral small-molecule versus injectable peptide GLP-1 receptor agonists within contemporary diabetes treatment algorithms [15,25,30,31,37,46,49,56,58,63,74,78,92,93].

**Table 8:** Comparative positioning of oral small-molecule versus injectable peptide GLP-1RAs in type 2 diabetes care GLP-1RA, glucagon-like peptide-1 receptor agonist; HbA1c, glycated hemoglobin; SGLT2, sodium-glucose cotransporter-2

Clinical consideration	Oral small-molecule GLP-1RAs	Injectable peptide GLP-1RAs
Typical placement in treatment algorithm	May facilitate earlier initiation in patients reluctant to inject	Commonly recommended when GLP-1RA therapy is clearly indicated
HbA1c-lowering expectations	Moderate to strong (agent- and dose-dependent)	Strong and well-established across multiple agents
Weight effect	Clinically meaningful weight reduction anticipated	Moderate to substantial weight loss demonstrated in trials
Cardiovascular risk modification	Outcome evidence evolving	Proven cardiovascular benefit for several agents
Use with metformin	Compatible and commonly anticipated	Compatible and guideline-endorsed
Use with SGLT2 inhibitors	Potential additive metabolic benefits	Established complementary use
Use with insulin	Requires further evidence for insulin-sparing effects	Proven insulin-sparing and weight-mitigating effects
Adherence considerations	Daily oral dosing; dependent on patient routine	Weekly dosing improves convenience but requires injection
Primary care adoption	May reduce therapeutic inertia	Adoption limited by injection aversion in some settings

Comparative Implementation in Obesity Medicine

Obesity is a chronic relapsing disease; long-term pharmacotherapy is often required to maintain weight loss [36,80]. Injectable agents have set high-efficacy expectations, but access and persistence remain limiting factors at the population scale [58,95]. Oral small-molecule GLP-1RAs could improve scalability by simplifying distribution and potentially lowering total delivery costs [62,94]. Daily oral therapy might reduce stigma for some patients who associate injections with “severe disease” [56,96]. However, adherence challenges in asymptomatic chronic conditions can be substantial, and daily pills are not automatically easier than weekly injections [63,97]. Weight-loss outcomes should be evaluated not only at 68-72 weeks but also across multi-year horizons to assess durability and safety [36,98]. Co-management with lifestyle, sleep, and mental health interventions remains essential because behavioral and environmental drivers persist [38,80]. If oral small-molecule agents achieve even moderate long-term weight loss with high persistence, even if patients do not reach a predefined target weight, they could meaningfully reduce cardiometabolic disease burden at scale if patients maintain continued therapy over time, supporting durable weight loss [15,99].

Given the chronic and relapsing nature of obesity, treatment selection must balance efficacy, scalability, and long-term adherence; these practical considerations for oral and injectable GLP-1 receptor agonist strategies are outlined in Table 9 [15,33,34,36-38,56,58,63,80,94-96,98,99].

**Table 9:** Role of oral and injectable GLP-1RA strategies in chronic obesity management GLP-1RA, glucagon-like peptide-1 receptor agonist; GI, gastrointestinal

Domain	Oral small-molecule GLP-1RAs	Injectable peptide GLP-1RAs
Route acceptability	High for patients preferring non-injectable therapy	Lower in injection-averse individuals
Weight-loss magnitude	Moderate to potentially high, depending on the agent	High to very high with established agents
Scalability at the population level	Potentially high due to oral administration	Limited by cost, access, and injection logistics
Long-term adherence	Dependent on daily dosing consistency	Supported by weekly dosing but affected by tolerability
Stigma perception	May reduce stigma associated with injectable therapy	Some patients perceive injections as “advanced disease”
Discontinuation risk	Driven by GI intolerance or daily pill fatigue	Driven by GI intolerance, supply interruptions
Weight regain after cessation	Expected without ongoing therapy	Expected without ongoing therapy
Integration with lifestyle therapy	Compatible with behavioral and dietary programs	Compatible with structured weight-management programs
Health equity implications	May improve access in resource-limited settings	Access limited by coverage and distribution constraints

Special Populations and Clinical Nuances

Older adults may benefit from weight loss and glycemic control but require careful monitoring for sarcopenia risk and nutritional adequacy [100]. Patients with advanced CKD may have limited medication options; GLP-1RAs are valuable, but agent-specific labeling and tolerability matter [30,24]. Hepatic impairment may alter clearance for small molecules more than peptides, potentially requiring dose adjustments or monitoring [22,24]. Women of reproductive age require counseling regarding pregnancy planning because weight-loss pharmacotherapy is generally avoided during pregnancy [101]. Patients with prior pancreatitis or gallbladder disease require individualized risk discussion and monitoring strategies [52,53]. For patients with significant diabetic retinopathy, gradual glycemic improvement and ophthalmologic monitoring may be prudent with potent agents [55]. Perioperative/peri-procedural management should consider gastric-emptying effects and aspiration risk, with evolving consensus guidance [88,102]. Psychiatric comorbidity and eating disorders require careful screening, as appetite-modifying therapies can interact with mental health trajectories [103].

Patient-specific factors and comorbid conditions can significantly influence the safety and appropriateness of GLP-1 receptor agonist therapy; Table 10 highlights key considerations across selected special populations [24,41,47,51-53,55,69,83,88,100,101,103].

**Table 10:** Special population considerations for oral and injectable GLP-1RAs GLP-1RA, glucagon-like peptide-1 receptor agonist; CKD, chronic kidney disease; HbA1c, glycated hemoglobin

Population/scenario	Oral small-molecule GLP-1RAs	Injectable peptide GLP-1RAs
Older adults	Requires monitoring for frailty and sarcopenia	Similar considerations; dose titration essential
Chronic kidney disease	Agent-specific metabolism may influence dosing	Generally safe across CKD stages with labeling guidance
Hepatic impairment	Greater relevance due to hepatic metabolism	Less dependent on hepatic clearance
Prior pancreatitis	Use with caution; limited long-term data	Use with caution; larger safety experience available
Gallbladder disease	Monitor if significant weight loss occurs	Similar risk, particularly with rapid weight reduction
Diabetic retinopathy	Gradual HbA1c lowering advised	Same precaution with potent glycemic effects
Peri-procedural management	Assess gastric-emptying effects	Similar gastric-emptying considerations
Polypharmacy	Evaluate drug-drug interaction potential	Minimal interaction risk
Pregnancy planning	Generally avoided; counseling required	Generally avoided; counseling required

Economic and Health System Considerations

The clinical impact of GLP-1RAs is constrained by affordability, prior authorization burden, and intermittent coverage changes [58,94]. Manufacturing and distribution differences between peptides and small molecules may influence future pricing, but this remains uncertain [62,104]. From a payer perspective, the value proposition improves if therapies demonstrably reduce cardiovascular events and downstream costs [39,105]. Therefore, cardiovascular outcome data for small-molecule oral GLP-1RAs will be pivotal for broad reimbursement in high-risk populations [65,105]. Workflows that reduce discontinuation (education, side-effect management, and follow-up) can improve cost-effectiveness by increasing time on therapy [59,90]. Health equity considerations are central because obesity and diabetes disproportionately affect underserved communities with limited access to specialty care [106]. Oral therapies may reduce access barriers related to injection training and cold storage, potentially improving equity [57,62]. However, if oral agents remain expensive or tightly restricted, theoretical advantages may not translate into population benefit [58,94].

Future perspectives: Where the field is heading

Next-generation incretin therapies include oral small-molecule GLP-1RAs, oral peptide strategies, and multi-agonists (GLP-1/GIP/glucagon combinations) [107]. Multi-agonist biology aims to enhance weight loss and metabolic outcomes beyond single-pathway GLP-1 receptor activation [34,107]. Novel formulations may enable less frequent oral dosing or improved tolerability through modified release and optimized PK profiles [108]. Precision medicine approaches may match patients to agents based on tolerability phenotype, metabolic profile, and comorbidity targets (CV, liver, and kidney) [81,109]. Non-weight endpoints such as fatty liver disease improvement and sleep apnea outcomes are increasingly important in regulatory and clinical decision frameworks [110]. The integration of digital tools (connected scales, symptom trackers, and adherence reminders) may improve persistence and allow safer titration [90,111]. Comparative head-to-head trials and pragmatic real-world studies will be needed to determine whether oral small molecules can match injectables in effectiveness [70,74]. Ultimately, the most impactful innovation may be the therapy that combines high efficacy with scalable access and durable long-term use [15,94].

To contextualize ongoing development efforts and remaining evidence gaps, Table 11 highlights key milestones required for small-molecule oral GLP-1RAs to meaningfully alter clinical practice [39,47-49,65,70,74,86,94,105].

**Table 11:** Evidence milestones required for the clinical adoption of small-molecule oral GLP-1RAs GLP-1RA, glucagon-like peptide-1 receptor agonist; CVOT, cardiovascular outcomes trial; MACE, major adverse cardiovascular events; HbA1c, glycated hemoglobin; T2D, type 2 diabetes; eGFR, estimated glomerular filtration rate

Evidence milestone	Why it is required	What would be convincing
Head-to-head versus injectable GLP-1RA	Establishes comparative efficacy and tolerability	Non-inferior HbA1c + clinically meaningful weight loss with acceptable GI profile
Cardiovascular outcome trial (CVOT)	Drives guideline priority and payer access	Demonstrated MACE reduction in high-risk T2D and/or obesity populations
Kidney outcome characterization	Helps position in CKD phenotypes	Reduced albuminuria and slowed eGFR decline beyond glycemic effects
Real-world persistence studies	Determines population-level impact	Sustained time on therapy and dose maintenance over 12-24 months
Safety surveillance	Detects rare/idiosyncratic adverse events	Stable hepatic safety, pancreatitis/gallbladder signal clarity, and consistent labeling

## Conclusions

Injectable peptide GLP-1RAs have established strong efficacy for glycemic control, weight loss, and cardiovascular risk reduction, but practical barriers limit uptake and long-term persistence in many patients. True small-molecule oral GLP-1RAs represent a major innovation that could broaden access and simplify chronic therapy, yet the key clinical question is whether they can achieve injectable-like benefits with acceptable tolerability and durable adherence. As cardiovascular outcome data and real-world effectiveness evidence mature, oral small-molecule GLP-1RAs may reshape the incretin landscape by enabling earlier, wider, and more persistent use and potentially translating pharmacologic promise into population-scale cardiometabolic benefit.
